# Gray matter volume alterations in subjects with overweight and obesity: Evidence from a voxel-based meta-analysis

**DOI:** 10.3389/fpsyt.2022.955741

**Published:** 2022-09-26

**Authors:** Lei Li, Hua Yu, Ming Zhong, Siyi Liu, Wei Wei, Yajing Meng, Ming-li Li, Tao Li, Qiang Wang

**Affiliations:** ^1^Mental Health Center, West China Hospital, Sichuan University, Chengdu, Sichuan, China; ^2^Sichuan Clinical Medical Research Center for Mental Disorders, Chengdu, China; ^3^Department of Sport and Health Science, University of Exeter, Exeter, United Kingdom; ^4^Department of Neurobiology, Affiliated Mental Health Center & Hangzhou Seventh People’s Hospital, Zhejiang University School of Medicine, Hangzhou, Zhejiang, China

**Keywords:** overweight and obesity, BMI, gray matter volume, voxel-based morphometry, meta-analysis

## Abstract

**Background:**

Obesity is a multi-systemic disease with complex etiology. And consistent evidence indicated obesity or overweight subjects render brain structure changes. Increasing evidence indicates these subjects have shown widespread structural brain gray matter volume (GMV) changes. However, results from other neuroimaging studies have been inconsistent. Consequently, the question remains whether body mass index (BMI), a gold standard to define obesity/overweight, is associated with brain structural changes.

**Methods:**

This study will apply an updated meta-analysis of voxel-based GMV studies to compare GMV changes in overweight and obese subjects. Online databases were used to build on relevant studies published before May 2022. The updated Seed-based d Mapping with Permutation of Subject Images (SDM-PSI) explores GMV changes in individuals with overweight and obesity and further examines the correlation between GMV and obesity-related variables, specifically body mass index (BMI).

**Results:**

This research included fourteen studies and provided a whole-brain analysis of GMV distribution in overweight and obese individuals. It revealed lower GMV in brain regions, including the left putamen and right precentral gyrus, in individuals with overweight and obesity compared to lean controls. Further, meta-regression analyses revealed GMV in the left middle occipital gyrus was negatively correlated with the BMI of the whole sample.

**Conclusion:**

GMV decreased was reported in reward circuit processing areas and sensorimotor processing areas of individuals with overweight and obesity diagnoses, suggesting an underlying structural basis for reward processing and sensorimotor processing dysregulation in overweight and obese subjects. Our results also suggest that GMV in occipital gyrus, a key region for food visual and gustatory encoding, is negatively associated with BMI. These results provide further evidence for the dysregulated reward circuit in individuals with overweight and obesity.

## Introduction

The prevalence of obesity diagnoses has been rising rapidly, as an estimated of 650 million people worldwide are currently identified as medically overweight. Kowning that obesity is highly correlated with other issues, including an increased risk of type 2 diabetes, respiratory problems, cardiovascular disease, mood-related disorders, and negative effects on one’s quality of life, it is considered one of the leading factors of death (1). Despite the understanding of the harmful effects of obesity is increasing, the etiopathology of weight gain and obesity has remained unclear. Research suggests that the impairment of psychological circuits, such as the reward system which negatively reinforces food craving, can motivate people to engage in food-seeking, overeating, and other forms of substance abuse (2–7). Such responses are particularly pronounced in subjects who are overweight and obese (8).

Compensatory overeating might be one of the leading causes to obesity (maybe the most frequent). To understand the brain structural changes of compensatory overeating and BMI increase, neuroimaging has been used to compare subjects with obesity and healthy controls (HCs) in structural magnetic resonance imaging (MRI) analyses (9, 10). In patients with obesity, reduced regional gray matter volume (GMV) is frequently reported in precentral gyrus, medial prefrontal cortex, inferior frontal gyrus, and cerebellum, while the left middle frontal gyrus, cuneus, and inferior occipital gyrus showed an increase in GMV (9, 10). Other studies imply that the striatum plays a specific role in obesity as presenting high-calorie foods to individuals with an obesity diagnosis leads to greater activation in reward processing areas including caudate, putamen, amygdala, and orbitofrontal cortices, cognitive control related-areas including the prefrontal cortex and anterior cingulate cortex, and sensorimotor processing areas, particularly precentral gyrus (11–14). Research by Rothemund et al. (15) reported that, in overweight and obese subjects, decreased activation in striatal areas during food consumption compared with striatal enhanced activation to high-caloric food-related cues might be the reason for overeating and consequent weight gain (16). Therefore, striatal hypo-activity in response to food intake is considered to be one of the primary mechanisms of compensatory overeating and BMI increase (17, 18). The striatal region is a dopamine-rich region in the brain and thus a key hub of the reward circuit. Patients with obesity have a substandard availability of dopamine D2 receptors in the striatum, which could lead to an increased inability to control overeating impulses (19). Moreover, while body mass index (BMI) is deemed the gold standard for measuring obesity, BMI negatively correlates with GMV in the striatum(e.g., caudate, putamen), emphasizing BMI may be an important risk factor for GMV decreased (20). However, the previous systematic reviews and large-scale evidence are insufficient for exploring GMV changes related to obesity.

Consequently, different from the Anisotropic Effect-Size Seed-Based d Mapping (AES-SDM) (9, 10) and Activation likelihood estimation (ALE) (21), we have taken an innovative approach, a newly released version of Seed-based d Mapping with Permutation of Subject Images (SDM-PSI), to provide a better understanding of the GMV changes in subjects with overweight and obesity. According to the developer’s statement, it uses a standard permutation test to evaluate whether effects are null or not and generates more reliable results than previous SDM-meta versions. This method not only avoids the drawbacks of alternative procedures used in current coordinate-based meta-analyses methods (22, 23), but also obtains the following advantages: (a) accounting for both increases and decreases of the outcomes so that contradictory findings cancel each other (24); (b) using random-effects modeling to estimates effect size and thus guaranteeing reliability (25); (c) using subject-based permutation tests equal to those of FSL “randomized” tools (26); (d) using a threshold-free cluster enhancement (TFCE) statistics (27). Considering that BMI is not normally distributed in overweight/obesity and lean individuals, we will primarily focus on the group comparison of the GMV alterations between individuals with overweight and obesity and control subjects who are considered lean. The newest meta-analysis was published in 2018 and did not take the new meta-method. In these meta-analysis, the author has reported the GMV loss in left, middle, and right inferior frontal gyrus (including the insula), precentral gyrus, temporal cortex, and the cerebellum, and increased GMV in the left middle frontal gyrus, left cuneus, left inferior occipital gyrus, in overweight and obesity individuals compared with HCs (9). However, it did not analyze the correlations between GMV alterations and obesity-related variables such as BMI any further. For this study, we found only two relevant meta-analyses and reviews (74 percent of the articles were the same) that explored the negative correlations between GMV in the medial prefrontal cortex, left temporal pole, bilateral cerebellum and right orbitofrontal cortex and BMI in patients with obesity (10, 21). In addition, they included the studies as follows: waist circumference (28) and eating behavior (29) rather than BMI associated with GMV alternations, BMI-related regional GMV reductions in first-episode mania patients (30), major depression (31) and adolescent (32), and etc. The mixed sample size may confound the group comparison results. Thus, in the current studies, we excluded all studies above. Finally, the studies we included provided mean BMI, from the range of BMI in the original study, we see that overweight individuals were also included in the obese subjects, thus, we named all participants as overweight/obesity individuals.

In this meta-analysis, we will use the voxel-based meta-analysis via the novel algorithm (e.g., PSI) to identify morphometric changes in overweight and obese subjects compared with lean subjects. Secondly, we will analyze the correlation between GMV difference and BMI among the sample pool. With regard to changes in brain structure for individuals with overweight and obesity, we include voxel-based statistical analysis to compare local differences in GMV after spatial normalization is taken into account (33). From the literature reviewed above, based on models of addiction that habitual overeating leading to weight gain and obesity marks the progressive change of the striatum (34–36), we hypothesize that, consistent with previous research, GMV of the overweight and obese group will significantly decrease, especially in reward processing areas, such as putamen, and sensorimotor processing areas such as precentral gyrus, Secondly, we hypothesize that there is a correlation between BMI, as an obesity-related indictor, and GMV loss.

## Materials and methods

### Data source

Systematic and comprehensive searches of the PubMed^1^, Embase^2^, CENTER (Cochrane Library)^3^ and Google Scholar^4^ databases were conducted based on studies published till May 2022. The included keywords are as follows: obesity; overweight; voxel-based morphometry; VBM; volumetry; morphometry; structural MRI or gray matter. In addition, manual searches were performed among the reference sections of the review articles and retrieved studies.

The inclusion criteria are as follows: (1) the study included subjects with obesity vs. lean controls; (2) using VBM to analyze whole-brain GMV differences of subjects with overweight and obesity; (3) the results reported statistical parametric maps and peak coordinates of the GMV alterations which were normalized into the Montreal Neurological Institute (MNI) or Talairach space (TL); (4) peer-reviewed studies; (5) all subjects provided informed consent; (6) participants aged ≥ 18. In addition, the authors of published studies were contacted by email when necessary information was not provided in the studies.

**FIGURE 1:**
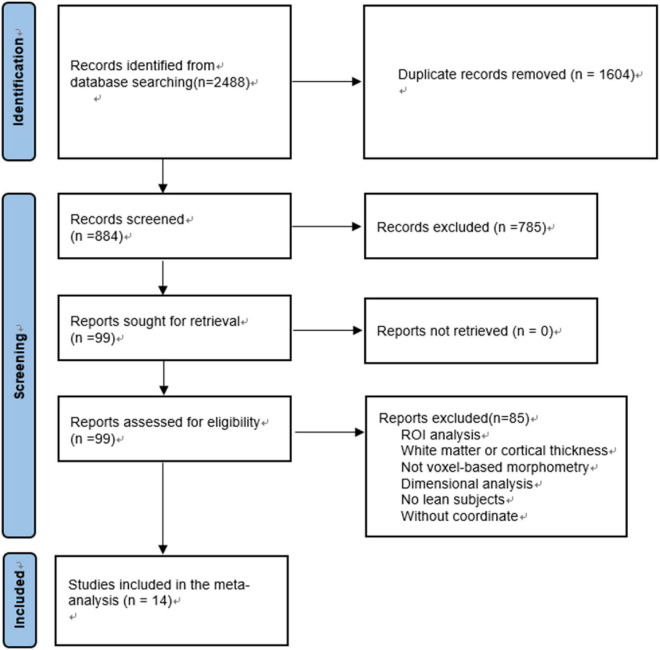
Flowchart of the selection of VBM studies in subjects with overweight and obesity for meta-analysis.

The exclusion criteria are as follows: (1) studies deal with seed voxel–based analysis, region-of interest (ROI), white matter changes or cortical thickness evaluations only rather than MRI whole-brain VBM; (2) review articles, theoretical papers, meta-analysis or animal experimental studies; (3) without lean controls; (4) participants aged<18; (5) when t- or z-maps were unavailable, consistent statistical thresholds throughout the brain were not used or peak coordinates were not reported (37) (Figure 1).

To evaluate the quality of perceived studies criteria was applied as follows: (1) lean controls compared with obesity/overweight subjects; (2) method of diagnosis; (3) demographic data; (4) samples size(When studies with sample size < 10, we scored as 0; sample size > 30, we scored as 2; the middle section is marked 1); (5) the use of GM volume covariates; (6) whole brain analysis; (7) MRI machines and smooth kernels (8) standard spatial coordinates (e.g., MNI coordinates or TL). Each criterion was independently estimated by two independent reviewers who scored as 0, 1 or 2 if the criteria were unfulfilled, partially met or fully met, respectively, and any study scoring > 8.0 was included in the meta-analysis. Although not specifically designed as an evaluation tool, this checklist provided an objective and strict indication of each study that included in our analysis (38).

### Voxel-wise meta-analysis

Regional differences in GMV between patients with overweight and obesity and lean individuals were analyzed with SDM-PSI^5^ (22). Details of the SDM-PSI method have been described previously (22). The procedure is briefly summarized as follows. First, the method of SDM-PSI combined the coordinates of cluster peaks with the effect sizes of significant differences between subjects with overweight and obesity and lean individuals to create whole-brain effect-size and signed maps, which were used to perform voxel-wise random effects meta-analyses (23, 38). Then, the SDM-PSI thresholds’ parameters were used in our analysis (full width at half maximum = 20 mm, voxel thresholds: TFCE CORRECTED > p, peak height thresholds: peak TFCE CORRECTED > 0.0500, extent threshold of clusters size > 1 voxels) (22). Next, mean map was obtained in regional GMV between subjects with overweight and obesity and lean individuals by voxel-based calculation of the mean of the study maps, weighted by the square root of the sample sizes of each study, so that studies with large sample sizes contributed more to the final map (39); Furthermore, reliability was confirmed by jackknife sensitivity analysis to assess the reproducibility of the results. Heterogeneity analyses were used to determine significant, unexplained differences of studies. Egger tests were performed to identify the asymmetry of funnel plots to examine conflicting studies and potential publication bias (40). The aim of the meta-regression analysis was to explore BMI was associated with the pooled effect size of the GMV difference between overweight/obese and the lean subjects.

## Results

### The sample characteristics

The present research included 14 structural MRI studies on overweight and obesity based on the search strategy. In total, there were 361 subjects with overweight and obesity (males = 107; females = 254; mean age range: 15.0 – 70.0 years; BMI rang: 26.20 – 43.10) and 419 controls (male = 188; female = 231; mean age range: 16.1–70.0 years; BMI rang: 20.96–24.0). Table 1 illustrated the demographic information.

**TABLE 1:** Description of the demographic and clinical characteristics in overweight and obesity subjects and lean subjects in the meta-analysis.

Study	Overweight and obesity subjects				Lean subjects				Magnetic field	Software	Smooth kernel
	Sample/ Female	Age (mean/SD)	BMI	Hand (left/right)	Sample/ Female	Age (mean/SD)	BMI	Hand (left/right)			
Brooks et al. (77)	59/34	70	33.0(0.3)	NA	97/52	70	22.5(0.2)	NA	1.5	SPM	8
Haltia et al. (78)	30/18	37(12)	33.0 (4.3)	NA	16/8	37 (21)	22.2 (1.6)	NA	1.5	SPM	12
Honea et al. (79)	72/49	38.9 (8.2)	35.6 (3.6)	NA	22/18	36.8 (10.9)	21.6 (1.6)	NA	3.0	SPM	10
Jauch-Chara et al. (80)	15/0	24.7(0.66)	36.3(1.04)	NA	15/0	24.6(0.69)	23.2(0.38)	NA	3.0	SPM	8
Karlsson et al. (81)	23/18	47.30 (8.90)	43.1(3.74)	NA	22/15	46.45 (9.45)	24.0(2.28)	NA	1.5	SPM	10
Mathar et al. (82)	19/8	27.0	33.6	(0/19)	23/12	25.1	21.8	(0/23)	3.0	SPM	8
Nouwen et al. (83)	13/12	15.0(1.9)	NA	NA	20/14	16.1(1.9)	NA	NA	3.0	SPM	6
Pannacciulli et al. (43)	24/13	32(8)	39.4(4.7)	NA	36/11	33(9)	22.7(2.2)	NA	1.5	SPM	8
Schienle et al. (84)	21/21	22.90 (2.59)	28.30 (3.40)	NA	21/21	22.57 (2.69)	22.34 (1.93)	NA	3.0	SPM	8
Shott et al. (85)	18/18	28.67 (8.30)	34.78(4.44)	NA	24/24	27.42 (6.28)	21.64(1.26)	NA	3.0	SPM	8
Smucny et al. (86)	28/14	30.29(3.81)	26.19(2.90)	NA	25/12	31.32(3.45)	20.96(1.99)	NA	3.0	SPM	8
Tuulari et al. (87)	47/42	44.9 (9.0)	42.2(4.0)	NA	29/23	45.9 (11.8)	23.2(2.8)	NA	1.5	SPM	8
Wang et al. (88)	31/7	39.58 (1.93)	34.38 (0.69)	(5/26)	49/21	29.55 (1.41)	21.87 (0.29)	(7/42)	3.0	SPM	8
Zhang et al. (89)	20/0	20∼28	33.56(3.53)	NA	20/0	20∼28	21.48(1.43)	NA	3.0	SPM	8

BMI: body mass index; SPM: statistical parametric morphometry.

### Regional gray matter volume differences

**FIGURE 2:**
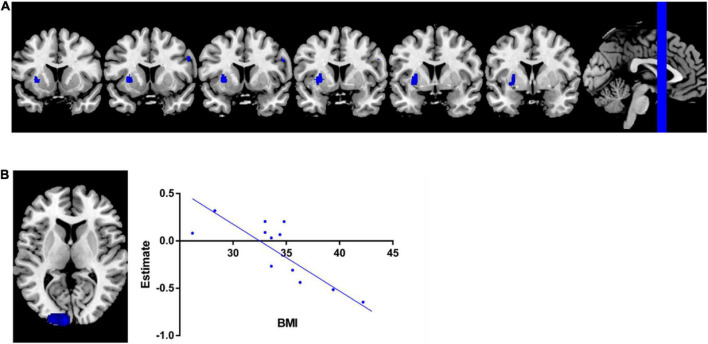
Meta-analysis results. **(A)** Regions showed lower GMV in overweight and obesity subjects than leans group. **(B)** Meta-regression analysis indicated that GMV in left middle occipital gyrus was significantly negatively associated with BMI in overweight and obesity individuals.

The pooled SDM-PSI meta-analysis map revealed significant lower GMV in subjects with overweight and obesity in the brain areas of the left putamen and right precentral gyrus (Figure 2A) with p-value less than 0.05 corrected by threshold-free cluster enhancement (TFCE) compared to lean group. Table 2 shows the peak coordinates and the cluster breakdown. No brain areas with increased GMV in individuals with overweight and obesity were observed in the present study.

**TABLE 2:** Lower gray matter volume in subjects with overweight and obesity compared with controls in the meta-analysis.

Anatomical regions	MNI coordinates x, y, z	SDM-z value	P* value	Number of voxels	Jackknife sensitivity
obesity < controls					
Left lenticular nucleus, putamen	−28,6,2	−5.004	0.0019	182	14/14
Right precentral gyrus, BA 44	52,8,30	−4.848	0.023	13	13/14

MNI = Montreal Neurological Institute; SDM = signed differential mapping; BA = Brodmann area; * The p-value was adjusted via threshold-free cluster enhancement(TFCE) (*p* < 0.05).

### Meta-regression analysis

The results of the meta-regression analysis revealed that GMV in the left middle occipital gyrus (MNI coordinate: −22, −98, 10; 180 peak voxels; SDM z = - 3.745; *p* = 0.00099) yielded a significant negative correlation with BMI of the current samples (Figure 2B), with adjustment *p*-value less than 0.05 corrected by using of TFCE statistics.

### Sensitivity, heterogeneity and publication bias

As shown in Table 2, the whole-brain jackknife sensitivity analysis of the meta-analysis revealed that GMV reduction in the left putamen was highly replicable, as this result was preserved when each study was removed. GMV decreased in the right precentral gyrus remained significant in 13 out of 14 combinations. Heterogeneity analysis results were reflected by the funnel plot and Egger tests, and the funnel plots did not reveal any publication bias (*p*:0.763 > 0.05) (see Supplementary Materials, Supplementary Figure 1).

## Discussion

This meta-analysis study revealed GM reduction in overweight and obese individuals, using a novel meta method of SDM-PSI based on 14 VBM studies. The results indicated lower GMV in the left putamen and right precentral gyrus in overweight and obese individuals in comparison with lean subjects, which is partly consistent with previously published meta-analysis studies (9). Moreover, for the purpose of this study, previous results were replicated, indicating that GMV in the left occipital gyrus was negatively associated with BMI in overweight and obese subjects (41, 42).

### Gray matter volume decreased in the putamen and precentral gyrus

This study revealed the reduced GMV in overweight and obese individuals in the putamen and precentral gyrus, in comparison with lean subjects, which aligns with previously published reviews (2, 9). The putamen is an important part of the striatum, and its structural change has been consistently observed in other empirical studies (43, 44). Accordingly, the putamen is understood to be able to code behavioral contingencies to obtain a specific reward with abundant dopamine D2 receptors (D2R), and overeating reduces dopamine D2R density, D2R sensitivity as well as reward sensitivity to food. Specifically, the morphometric decline in putamen is accompanied by the lower D2R availability among overweight and obese individuals (45). As a result, the reduced D2R availability in the striatal reward circuitry may result in increasing food craving compensatory and the resultant weight gain (46). In addition, previous studies also demonstrate that higher BMI was associated with down-regulated D2R in obese individuals (47), and correlated with the putamen hypo-activity in response to receipt palatable food in obese/overweight subjects (18). Consequently, the change in dopamine neuro-circuitry of the striatum may increase their susceptibility to opportunistic overeating while making food intake less rewarding (47).

In addition to the dopamine reward circuit, the putamen also performs a key role in the highly salient information processing, and is also involved in the origination, generation, and sequencing of motor behaviors (48). Consequently, the ability of information processing and behavior control would be limited by the development of putamen GMV changes (49), namely in the way that it develops an imbalance between autonomic processing and reward processing to overeat when food stimuli are present (49). Specifically, GMV loss in the putamen region would reduce the ability of self-control. The relationship between GMV loss in putamen, the reward circuitry, and behavior control may result from the reduced functional activation observed in the brain region.

Moreover, the function of the putamen is suggested to be lateralized (50, 51). That is, in healthy individuals, D1R, D2R, and D3R binding and dopamine synthesis capacity are higher on the right putamen than the left putamen (52), whereas the binding between the D1R and the dopamine transporter is higher in the left putamen than the right putamen (53). One of the potential hypotheses for such selective lateralization is suggested to be related to handedness, as putamen plays a key role in motor execution (54). Accordingly, the phenomenon might be related to handedness/hemispheric dominance. There is empirical evidence supporting this hypothesis. Research by Jang et al. (55) found that GMV in right putamen are larger in the left-handed subjects than in the right-handed subjects. As a result, the influence on putamen and behavior control would more salient to the left putamen in right-handed subjects. However, this deduction remains theoretical because the current analysis found little information on the patients’ handedness. The specific GMV reduction in the left putamen may diverse when patients have different dominate hands.

Additionally, this study reported consistent GMV loss in the right precentral gyrus (PCG). PCG controls motor activity and involves the execution of the elaborative motor activity (56). The recent studies have suggested that the relationship between motor control and obesity may be influenced by participants’ habits such as eating style (57) and preferences (58). Obese subjects took less time to plan the movement and more time to perform the movement in the face of more preferred food, and with worse motor control compared with lean subjects (59). The areas in PCG forming the sensorimotor networks (SMN) were understood to integrate information with the executive functions, perception, and somesthesis (60). Thus, the GMV reduced in PCG in overweight/obese subjects will have an effect on their ability to intergrade the perceived information, particularly in relation to perceptions of the size, weight, and shape of food, and further influenced their self-control to consume the food (61, 62). This may explain why obese patients are more likely to display overeating behavior and develop obesity. Thus, sensorimotor control might be considered a key pathology in overweight and obesity individuals. A pronounced correlation between sensorimotor and reward-related areas has been widely identified in obesity-related studies and further demonstrated that neural activity in sensorimotor regions is more dependent on reward-related regions. The reward-related regions, such as the putamen and caudate, interconnected with the primary motor cortex (precentral gyrus), which perform a fundamental role in sensorimotor control (63). In addition, the previous studies have estimated that it is possible that variations in total intracranial volume (64) and PCG (65) supporting sensorimotor control precede the development of overeating and obesity. Moreover, neuroanatomical differences may be a consequence of obesity. A longitudinal study found that sustained high BMI relates to greater progressive declines in GMV over 5 years, and obesity may contribute a vicious cycle of overeating behaviors (66). Specifically, the current meta-analysis has shown that loss GMV in PCG in obesity individuals, a region related to sensorimotor control. Reduced GMV in this area could hypothetically relate to limited control over food intake, leading to increased overeating. In turn, the accumulation of body fat caused by overeating might cause further structural damage in PCG.

This study identified deficits of GMV in the putamen and PCG, which is consistent with the previous meta-analysis studies (9), suggesting that areas in the putamen and PCG are the potential neuroanatomical biomarkers in overweight/obese subjects. However, GMV loss in the frontal gyrus, temporal cortex, and subcortical area was unconfirmed. One possible reason is that the observed group effect disappeared with the increased sample size, suggesting that GMV loss of these brain regions cannot be stable markers of overweight/obese subjects.

### Negative association between BMI and occipital gyrus

In the meta-regression analysis section of this study, it was ascertained that GMV in the left middle occipital gyrus was negatively associated with the BMI of the whole sample, indicated that BMI was negatively related to the pooled effect size of the GMV difference between overweight/obese subjects compared to the lean subjects in the left middle occipital gyrus, suggesting that higher BMI was related to greater GMV reduction in the left middle occipital gyrus, which is similar to findings in previous studies (41, 67). The occipital gyrus is one of the key brain areas involved in the neural processing of visual food stimuli in overweight and obese individuals (68). Similarly, empirical studies suggested that significant thinning of the cortex in the occipital gyrus is associated with increasing BMI (41, 42). Kullmann et al. (69) reported that increased body weight in overweight and obese individuals has the potential to change neural processing in high-level visual areas, such as the occipital cortex. Furthermore, the occipital gyrus also performs a key role in object recognition, which can rapidly discern the energetic value of food in relation to salience tracking of high-energy and low-energy food (70). As excess energy intake may contribute to a hyper-responsivity of reward and gustation regions, as a prompt for food intake, this suggests that the obese/overweight subjects are more likely to overeat (71). However, the result of the meta-regression is different from the results of the between-group comparison that GMV decreased in the putamen and precentral gyrus in the obese/overweight subjects. The possible reasons are that many other variables might influence the results, such as gender (44, 72), age (73), body fat percent (74), participants’ habits (75), possible psychiatric comorbidities (31). In addition, the low number of studies in literature fulfilling the eligibility criteria was also an important factor. Further studies are needed to elucidate brain morphological structure in overweight/obesity subjects.

## Limitations

It is important to highlight several limitations of this study. Firstly, the data is based on collated analysis which has been extracted from published studies, as opposed to the original data, which may result in less accurate results (76). More importantly, the results of the meta-regression analysis study and the results of the subgroup analysis for the obesity group and lean group (e.g., 11 studies reported brain structure differences between the obesity group (BMI > 30) and lean group, and 2 studies reported overweight group (25 < BMI < 30) and lean group, while one study did not clearly report the BMI range) should be considered carefully, as this study incorporated only a small number of studies that fulfilling the eligibility criteria and data availability was limited, future studies need to expand the sample size for further validation the GMV changes in subjects with overweight/obesity. Similarly, it was not possible to conduct a subgroup analysis of obesity grade, age, gender, and co-morbidity. Finally, longitudinal studies are necessary to examine whether the current brain structural changes in the putamen and precentral gyrus are causes or consequences of being overweight and obese.

## Conclusion

In summary, the present research reported the most robust structural reduced of the GMV in the putamen and precentral gyrus in overweight and obese individuals. Moreover, GMV in the left occipital gyrus was negatively associated with the BMI of our samples. Our results are replicated with previously published brain structural findings in overweight and obese subjects and suggest that these patients are accompanied with brain abnormalities.

## Data availability statement

The raw data supporting the conclusions of this article will be made available by the authors, without undue reservation.

## Author contributions

LL and HY were responsible for the study concept and design. HY, WW, YM, and M-LL collected the data, analyzed data, and interpreted the results. LL wrote the manuscript. MZ, SL, TL, and QW provided critical revision of the manuscript. All authors read and approved the final manuscript.

## Footnotes

1
https://www.ncbi.nlm.nih.gov/PubMed/

2
www.embase.com

3
https://www.cochrane.org/

4
http://scholar.google.com/

5
http://www.sdmproject.com/
