# Supplementary material for: Gray matter volume alterations in subjects with overweight and obesity: Evidence from a voxel-based meta-analysis
Source: Front Psychiatry. 2022 Sep 26;13:955741. doi: 10.3389/fpsyt.2022.955741 (PMC9548618; doi:10.3389/fpsyt.2022.955741)
Supplement: Supplementary file 1 [file Table_1.DOCX]

**Supplementary materials**

**Figure Legends**


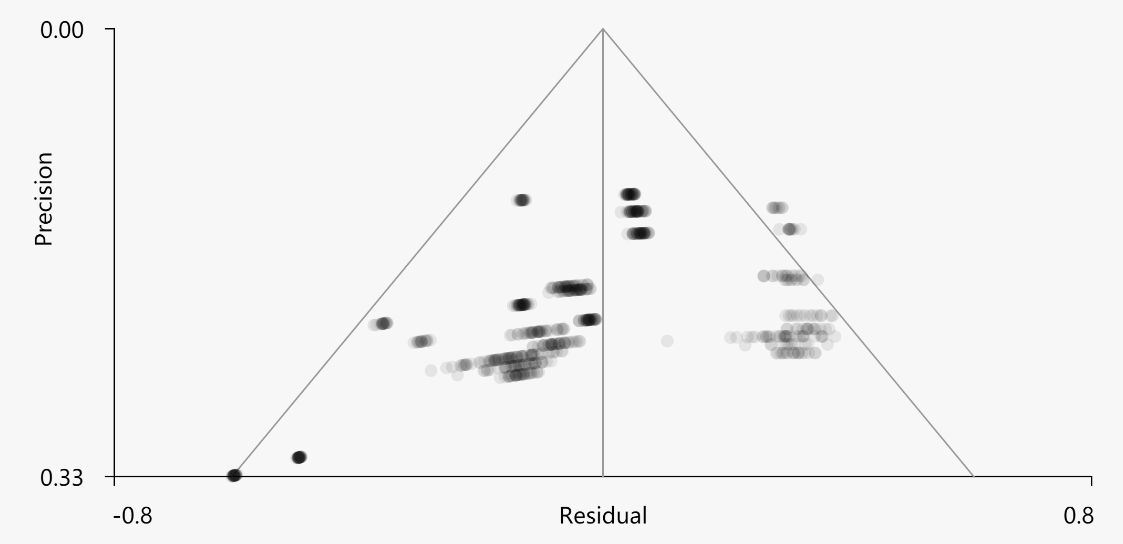


**Figure 1.** Funnel plots were used to assess publication bias. The funnel plot did not reveal any publication bias as Egger test > 0.05
